# Proceedings of the State Society

**Published:** 1856-01

**Authors:** 


### PROCEEDINGS OF THE STATE SOCIETY.

By a resolution of that body, it was made the duty of this Journal to publish the proceedings and send a copy to each member of the society. We *did* publish and we *did* send to each member as far as we were acquainted with each and his locality. But we did not know all, nor have we been able, as yet, to procure the Constitution, and with it, a list of the old members, although we have persevered with our efforts to this effect.

Those knowing themselves to be members and have not received a copy, will please give us notice, and it will be promptly sent. We have traced the Constitution and Bye-Laws to the possession of one of the former Secretaries, Dr. Ransom of Burlington, but we have heard nothing of the record since. Will Dr. Bird, the present Secretary, make some enquiries in that direction?

